# COMP Report: A survey of radiation safety regulations for medical imaging x‐ray equipment in Canada

**DOI:** 10.1002/acm2.12708

**Published:** 2019-09-20

**Authors:** Thorarin A. Bjarnason, Robert Rees, Judy Kainz, Lawrence H. Le, Errol E. Stewart, Brent Preston, Idris Elbakri, Ingvar A. J. Fife, Ting‐Yim Lee, I Martin Benoît Gagnon, Clément Arsenault, Pierre Therrien, Edward Kendall, Elena Tonkopi, Michelle Cottreau, John E. Aldrich

**Affiliations:** ^1^ Medical Imaging Interior Health Authority Kelowna BC Canada; ^2^ Radiology University of British Columbia Vancouver BC Canada; ^3^ Physics University of British Columbia Okanagan Kelowna BC Canada; ^4^ Occupational Health & Safety Yukon Workers' Compensation Health and Safety Board Whitehorse YK Canada; ^5^ Workers' Safety and Compensation Commission for Northwest Territories and Nunavut Yellowknife NT Canada; ^6^ Diagnostic Imaging Alberta Health Services Calgary AB Canada; ^7^ Radiology and Diagnostic Imaging University of Alberta Edmonton AB Canada; ^8^ Radiation Safety Unit Government of Saskatchewan Saskatoon SK Canada; ^9^ Cancer Care Manitoba Winnipeg MB Canada; ^10^ Physics and Astronomy University of Manitoba Winnipeg MB Canada; ^11^ Radiology University of Manitoba Winnipeg MB Canada; ^12^ St Joseph’s Health Care London London ON Canada; ^13^ Lawson Research Institute London ON Canada; ^14^ Medical Imaging Medical Biophysics, Oncology Robarts Research Institute University of Western Ontario London ON Canada; ^15^ Ministère de la Santé et des Services sociaux du Québec Québec Canada; ^16^ Hôpital Dr Georges–L. Dumont Centre d'Oncologie Dr Léon–Richard Moncton NB Canada; ^17^ Therapeutic Physics Horizon Health Network Saint‐John NB Canada; ^18^ Faculty of Medicine Memorial University St John’s NL Canada; ^19^ Nova Scotia Health Authority Halifax NS Canada; ^20^ Diagnostic Radiology Dalhousie University Halifax NS Canada; ^21^ Radiation Oncology Dalhousie University Halifax NS Canada; ^22^ Health PEI ‐ Diagnostic Imaging Charlottetown PEI Canada; ^23^ Radiology University of British Columbia Vancouver BC Canada

**Keywords:** diagnostic radiology, medical imaging, occupational dose, radiation safety, shielding

## Abstract

X‐ray regulations and room design methodology vary widely across Canada. The Canadian Organization of Medical Physicists (COMP) conducted a survey in 2016/2017 to provide a useful snapshot of existing variations in rules and methodologies for human patient medical imaging facilities. Some jurisdictions no longer have radiation safety regulatory requirements and COMP is concerned that lack of regulatory oversight might erode safe practices. Harmonized standards will facilitate oversight that will ensure continued attention is given to public safety and to control workplace exposure. COMP encourages all Canadian jurisdictions to adopt the dose limits and constraints outlined in Health Canada Safety Code 35 with the codicil that the design standards be updated to those outlined in NCRP 147 and BIR 2012.

## 1 INTRODUCTION

Radiation use in Canada is regulated by the Canadian Nuclear Safety Commission (CNSC) and Federal, Territorial, and Provincial governments. Radionuclides, above published exemption amounts, fall under the exclusive jurisdiction of the CNSC. The CNSC issues licenses for the manufacture, acquisition, and use of radionuclides and the standards are uniformly applied across the country. The situation is different for x‐ray‐emitting devices with energies below 1 MeV. Federal agencies authorize the sale, lease, and importation of x‐ray devices, but it is the jurisdiction of the Provinces and Territories to regulate the installation and use of medical x‐ray imaging equipment. Consequently, permitted uses, occupational dose constraints and limits, and shielding design of x‐ray facilities vary across the country. As many jurisdictions are attempting to reduce the legislative burden of radiation safety regulations, the Canadian Organization of Medical Physicists (COMP) conducted a survey in 2016/2017 to provide a useful snapshot of existing variations in rules and methodologies for human patient medical imaging facilities in order to assist jurisdictions to harmonize approaches.

## 2 BACKGROUND

Canada has adopted the guidelines of the International Commission on Radiological Protection (ICRP) on occupational dose limits for radiation. Starting with the publication of ICRP 26^1^ in 1977, estimates were given of the radiation sensitivities of various organs and tissues (*w_t_*), and the whole‐body dose was considered as the sum of doses to all organs and tissues each weighted for their radiation sensitivities. Publication ICRP 60 (1991)^2^ improved upon ICRP 26 with better data on radiation sensitivities. Equivalent dose (*H_R_*) was defined as the absorbed dose multiplied by a radiation weighting factor (*w_R_*) related to relative biological effect of a given type of primary radiation. For x‐ray photons of concern here, *w_R_* = 1. Effective Dose (*E*) was defined as the sum of the equivalent dose to each organ or tissue weighted by the relevant radiation sensitivity. ICRP 103 (2007),^3^ using new data, further refined the tissue sensitivities. The tissue weighting factors from the different ICRP reports are compared in Table 1 and it is noteworthy that these weighting factors change over time as the understanding of the effects of radiation on human biology improves.

**Table 1:** Tissue Weighing Factors (*w_t_*) from ICRP26, ICRP60, and ICRP103. The definition and handling of doses to Remainder tissues changed after ICRP26. Remainder tissues for ICRP103 are as follows: adrenals, extrathoracic region, gall bladder, heart, kidneys, lymphatic nodes, muscle, oral mucosa, pancreas, prostate, small intestine, spleen, thymus, and uterus/cervix.

Tissue or organ	***w_t_***		
	ICRP26 (1977)	ICRP60 (1991)	ICRP103 (2007)
Gonads	0.25	0.20	0.08
Bone Marrow	0.12	0.12	0.12
Colon	–	0.12	0.12
Lung	0.12	0.12	0.12
Stomach	–	0.12	0.12
Breast	0.15	0.05	0.12
Bladder	–	0.05	0.04
Liver	–	0.05	0.04
Esophagus	–	0.05	0.04
Thyroid	0.03	0.05	0.04
Skin	–	0.01	0.01
Bone Surface	0.03	0.01	0.01
Brain	–	–	0.01
Salivary glands	–	–	0.01
Remainder	0.30	0.05	0.12

At the time of publication for ICRP 103, the occupational limit for eyes was under review, and ICRP 118 was subsequently published recommending a lower limit for the eyes.^4^ The recommended stochastic dose limits from ICRP 60, 103, and 118 are shown in Table 2.

**Table 2:** Occupational and nonmedical stochastic dose limits from ICRP 60 103, and 118 for planned exposure situations.

Person	Dose limits		
	ICRP 60 (1991)	ICRP 103 (2007)	ICRP 118 (2012)
Radiation worker (effective dose)	20 mSv/yr — 5 yr average and not exceeding 50 mSv in one year	20 mSv/yr—5 yr average and not exceeding 50 mSv in 1 yr	–
Pregnant radiation worker	2 mSv for duration of pregnancy once declared (equivalent dose to the surface of the abdomen)	1 mSv for duration of pregnancy once declared (effective dose to the fetus)	–
Hands and feet of radiation worker (equivalent dose)	500 mSv/yr (localized exposure)	500 mSv/yr (averaged over 1 cm^2^ area of exposed skin)	–
Eyes (equivalent dose for occupational worker)	150 mSv/yr	150 mSv/yr	20 mSv/yr— 5 yr average and not exceeding 50 mSv in 1 yr
Members of the public (effective dose)	1 mSv/yr	1 mSv/yr	–

### 2.1 Dose constraints, diagnostic reference levels, and dose limits

A dose constraint is a restriction on the prospective doses to individuals that may result from a defined source of radiation, providing a basic level of protection for a population from planned exposure situations. The dose constraint is often chosen as a level of dose and do not apply to medical diagnosis or treatment. Diagnostic reference levels are similar to dose constraints, but are intended for medical exposure situations. An example of a dose constraint is shielding adjacent spaces of an x‐ray room to planned occupational dose levels. And an example of a diagnostic reference level is the 75th percentile of a distribution of dose indicator values used for a specific patient population for a given diagnostic exam. A dose limit is used for the occupational exposure of individuals, and is a limit of exposure from all occupational sources. Two examples of dose limits are full body exposure limits for radiation workers and eye exposure limits mostly of concern in fluoroscopy guided interventional (FGI) procedures. In both cases, if the annual dose limit is exceeded the individual should be prevented from receiving further occupational exposures for a year. Dose limits do not apply to patients.

### 2.2 Optimization

The dose limits in Table 2 were set at *the limit of unacceptability* in ICRP 60, where it was recommended that occupational exposure be kept as low as reasonably achievable (ALARA), taking social and economic considerations into account, in order to avoid exceeding these limits. These values were retained in ICRP 103. In the USA National Council on Radiation Protection & Measurements (NCRP) report 147 it was recommended that the shielding design constraint should be 5 mSv per year for radiation workers and 1 mSv for the general public.^5^

In the United Kingdom (UK), the National Radiological Protection board recommends that for optimization purposes the dose constraint should not exceed 30 % of the dose limit.^6^ This recommendation is in line with ICRP, which also recommends an occupational dose constraint of ≤20 mSv/yr.^3^ From personnel dosimetry it was found that the average dose to UK radiology technologists was 0.06 mSv/yr. As a result, the annual dose constraint of 0.3 mSv was adopted for workers in the shielding design of x‐ray facilities, as this dose constraint was already being met.^6^

### 2.3 Radiation shielding design

NCRP 49, published in 1976,^7^ was the main guide to x‐ray shielding in North America until the publication of NCRP 147 in 2004. NCRP 49 was essentially the same as NCRP 34^8^ first published in 1970. In the late 1980s, NCRP 49 came under a lot of criticism for lacking important information and being overly conservative, including^9^:

- No information on modalities such as computed tomography (CT), mammography, and digital imaging.
- Attenuation data were not applicable to three phase or constant potential generators.
- Typical mAs workloads were no longer valid due to the use of newer high speed rare‐earth film/screens.
- The use factors and occupancy factors appeared to be unrealistically high.
- Shielding was specified using half‐value‐layers (HVLs) of Pb or concrete required to attenuate scattered and primary radiation to designed levels, and the requirement to “add‐one‐HVL” was considered overly‐conservative.
- The requirement to cover screws or nails with Pb tabs was questioned.

Funding and other issues, however, hampered the publication of a new shielding guide until 2004. The shielding design recommendations of NCRP 147 addressed most of the shortcomings of NCRP 49 listed above. Instead of a formulaic approach to the calculation of primary, scatter, and leakage radiation, NCRP 147 lists actual field measurements for typical radiological examinations. Extensive attenuation data are given which can easily be incorporated in a spreadsheet or other software. Workload data are also taken from actual surveys across the USA. One paper that compared shielding design using NCRP 49 and NCRP 147 showed that NCRP 49 methodology overestimated the required thickness of Pb by up to 50%.^10^

NCRP 147 is not without criticism, however, including concerns about tertiary radiation scattered from the ceilings of CT and angiography rooms into control areas.^11^ For high workload rooms the dose to staff in adjacent rooms can exceed several mSv/yr.^11^

The British Institute of Radiology’s (BIR) *Radiation Shielding for Diagnostic x‐rays* published in 2000^12^ and updated in 2012^6^ uses a different approach to shielding calculation compared to the NCRP. Primary barriers are calculated assuming a standard dose is required at the detector whether this be film, computed radiography, or direct digital radiography. Scattered radiation barriers for radiography and FGI systems are calculated using a formula relating scattered dose to the tube voltage and dose indicator Kerma Area Product. Scatter in CT rooms is calculated from the dose indicator Dose Length Product for studies performed in the room. The 2012 version of the guide also covers tertiary scatter from ceilings and labyrinths. It is of interest that leakage radiation was considered a prominent type of radiation in NCRP 49 but is in fact completely ignored in the BIR publications.

In federal workplaces, Health Canada Safety Codes play an important role in radiation safety. Overall, federal departments are regulated by the Canada Labour Code (CLC) which includes requirements addressing workplace health and safety. More explicitly, promulgated under the CLC, the Canada Occupational Health and Safety Regulations (COHSR) set out the requirements of an employer, where a device that is capable of producing and emitting energy in the form of ionizing or nonionizing radiation is used in the workplace. Under the COHSR regulations, the requirement to implement prescribed safety codes (and safety standards) is outlined (Section 10.26, Note 1). Thus, Health Canada Safety Codes are primarily for the instruction and guidance of persons employed by federally regulated employers, or those under the jurisdiction of the CLC, and they are not themselves regulations, they are guidance documents; however, they can and do become a regulatory requirement when incorporated by reference into other regulations or acts — federal, provincial, or territorial.

Safety Code (SC) 20A^13^ was first published in 1976. Safety Code 20A was mainly concerned with safety procedures for the installation, use, and control of x‐ray equipment. It had limited sections on the x‐ray output parameters. Only film processor quality control was defined in any detail.

Safety Code 35 (2008)^14^ is a vast improvement on the former Code, and COMP endorses the provincial and territorial adoption of this code into jurisdictional regulations and accreditation agencies.^15^ Safety Code 35 includes comprehensive safety requirements for the installation, use, and control of all x‐ray equipment (except mammography equipment covered in SC 36,^16^ dentistry equipment covered in SC 30,^17^ and bone mineral density equipment). There is increased emphasis on patient dose and much of SC 35 is concerned with quality control (QC) of digital imaging systems. Unfortunately, although SC 35 explicitly states that radiation shielding should be designed using the methods of NCRP 147, an Appendix has a summary of NCRP 49 which was retained from SC20A at the request of some provincial radiation protection authorities for their reference.

## 3 MATERIALS AND METHODS

### 3.1 How the survey was conducted

The Canadian Organization of Medical Physicists endeavored to capture existing rules and methodologies for human patient medical imaging facilities. Participants from the COMP Imaging Committee, and their contacts representing every Province and Territory, were invited to provide information on the regulations governing the use of x‐rays, as well as related dose limits and constraints. The survey was performed in November 2016 and includes human patient medical imaging facilities only, and excludes installations within penitentiaries (Correctional Services Canada), Department of National Defence, First Nations and Inuit Health Branch and other federally regulated facilities.

### 3.2 Health Canada national dose registry data

Radiography occupational dose information was provided by Health Canada's National Dose Registry for the years 1990–2016, broken down by province and territory. Health Canada has published a report containing these data for Medical and other job sectors.^18^

## 4 RESULTS AND DISCUSSION

### 4.1 Regulations in each province/territory

Table 3 provides a summary of the provincial and territorial acts and regulations. All provinces and territories have x‐ray Acts and/or Regulations in place except New Brunswick, Nova Scotia, and Prince Edward Island. In New Brunswick the *Radiological Health Protection Act* was repealed in 2007. In Prince Edward Island *The Radiation Safety Regulations* were revoked on August 1, 2016, which COMP strongly opposed.^19^

**Table 3:** Provincial and territorial acts and regulations.

Province or territory	Regulations regarding radiation safety
Alberta	Radiation Protection Regulations (2003) under the Radiation Protection Act http://work.alberta.ca/occupational-health-safety/radiation-legislation.html
British Columbia	WorkSafeBC, College of Physician and Surgeon’s Diagnostic Accreditation Program. WorkSafeBC is obliged under their Regulations to enforce all Health Canada Safety Codes. https://www.worksafebc.com/en/law-policy/occupational-health-safety/searchable-ohs-regulation/ohs-regulation/part-07-noise-vibration-radiation-and-temperature; https://www.cpsbc.ca/programs/dap
Manitoba	The Radiation Protection Act. Regulations being drafted https://web2.gov.mb.ca/bills/40-4/b037e.php
New Brunswick	No regulations
Newfoundland and Labrador	Radiation Health and Safety Regulations under the Radiation Health and Safety Act (2003)
Northwest Territories and Nunavut	Occupational Health & Safety regulations, Part 23, Sections 339‐363
Nova Scotia	No regulations
Ontario	Healing Arts Radiation Protection Act, Ontario Regulation 543 and Regulation 861‐90 under the Occupation Health and Safety Act for workers
Prince Edward Island	No regulations
Quebec	Loi sur la santé publique Loi sur les services de santé et services sociaux Loi et Règlement sur les laboratoires médicaux, la conservation d’organes et les tissus et la disposition des cadavres Loi sur la santé et la sécurité du travail (RLRQ, chapitre S‐2.1) et son Règlement d’application.
Saskatchewan	The Saskatchewan Employment Act and The Radiation Health and Safety Regulations, 2005.
Yukon	Yukon Occupational Health and Safety Act.

In most provinces and territories, x‐rays are regulated under Labour or Health legislation and implementation and/or enforcement is performed by the provincial/territorial occupational health and safety departments, such as WorkSafeBC in British Columbia and Ministry of Labour Relations and Workplace Safety in Saskatchewan, for examples. In Manitoba x‐ray Inspectors have a letter of appointment from Minister of Health.

### 4.2 Dose limits

As shown in Table 4, many jurisdictions use the annual dose limits from SC 20A; that is, 50 mSv for x‐ray workers, 1 mSv for the public, and 4 mSv for the remainder of a pregnancy following declaration. A few provinces have adopted the more recent 20 mSv for radiation workers from SC 35, and some jurisdictions have no limits due to the lack of regulations. For jurisdictions without regulations, institutions or authorities usually set their own limits as best practice, but there is a risk they might not.

**Table 4:** Provincial and territorial breakdown of dose limits.

Province or territory	Dose limits		
	X‐ray Worker (mSv/yr)	General public (msv/yr)	Pregnant worker (mSv) c
Alberta	50^a^	1	4
British Columbia	20	1	4
Manitoba	20	1	4
New Brunswick	NA	NA	NA
Newfoundland and Labrador	50	5	5
Northwest Territories and Nunavut	50^a^	1	4
Nova Scotia	20	1	4
Ontario	50	1^b^	5
Prince Edward Island	NA	NA	NA
Quebec	50^a^	1	4^d^
Saskatchewan	50^a^	1	4
Yukon	NA	NA	NA

NA = not available.

a With an additional 5‐yr cumulative dose limit of 100 mSv.

b 5 mSv for other workers (non‐x‐ray).

c For the remainder of the pregnancy.

d Could be 2 mSv or lower depending on the designated regional occupational health physician.

### 4.3 Shielding of x‐ray facilities

No two provinces or territories have the same standards for the shielding of x‐ray facilities, as shown in Table 5. The responsibility for designing and subsequently verifying shielding installation varies widely, from being the responsibility of the owner (e.g., private clinics in Alberta), to a dedicated radiation protection group within government or the health care sector (e.g., CancerCare Manitoba). In some jurisdictions design templates are available (e.g., from the Radiation Safety Unit in Saskatchewan and the Centre for Disease Control in British Columbia, although the British Columbia entity no longer provides this service) and for rooms that do not satisfy the template criteria, other experts, such as those listed in Table 5 are used.

**Table 5:** Shielding design personnel by province and territory for radiological x‐ray, fluoroscopy, and computed tomography (CT) rooms.

Province or territory	Allowed to perform shielding design	Approves shielding design	Visual inspection required?	Scatter survey required after construction complete?
Alberta	Not Regulated	N/A	Yes. By ARPA inspector who did not design the room shielding	Yes. By an ARPA inspector who did not design the room shielding
Alberta Health Services	Medical physicists who are also Authorized Radiation Protection Agency (ARPA) inspectors	N/A	Yes. By ARPA inspector who did not design the room shielding	Yes. By an ARPA inspector who did not design the room shielding
British Columbia	“trained individuals with current in‐depth knowledge of structural shielding design” Note^9^	Not applicable	No	Yes
Manitoba	Radiation Protection Officers (with appointment as x‐ray Inspector)/Radiation Safety specialist	CCMB Radiation Protection Group ‐ Radiation Protection Officers (with appointment as X‐ray Inspector)/ designated CCMB Health Physicist or CCMB Medical Imaging Physicist	Yes (photo record kept)	No Sometimes performed in special circumstances
New Brunswick	Not regulated. Usually performed by medical physicist hired on contract	Not regulated. Usually performed by medical physicist hired on contract	No	No
Newfoundland and Labrador	Manufacturer, Medical Physicist, Engineer, etc	Compliance and Regulatory Affairs Officers, Occupational Health and Safety, Service NL	No	Yes
Northwest Territories and Nunavut	None specified	Chief safety Officer	No	No
Nova Scotia	Performed by Medical Physicist, not regulated	Typically Medical Physicist, not regulated	No	No
Ontario	Qualification not specified but in practice by someone approved by the Radiation Protection Service of Ontario	X‐ray Inspection Service via BCEP	No	No
Prince Edward Island	Medical Physicist	Medical Physicist	Yes	Yes
Quebec	Engineer; if necessary with the help of a qualified physicist	Private facilities: Laboratoire de Santé Publique du Québec (LSPQ) Public Facilities: no mandate; this falls the under engineer responsibilities when they stamp and seal a design	Private facilities: Yes Public Facilities: Yes	Private facilities: Yes, with report to LSPQ Public Facilities: Yes
Saskatchewan	Shielding Consultant if requirements outlined in shielding manual can’t be met	Radiation Safety Unit	No	No
Yukon	Not specified	X‐ray inspector on behalf of Director	Yes	No

In some jurisdictions shielding designs falls under the purview of engineers. Engineers Canada publishes national guidelines on the practice of engineering in Canada, with input from all provincial and territorial associations, which may be adopted in part, in whole, or not at all by engineering regulators in Canada. This organization defines the practice of engineering as “any act of planning, designing, composing, evaluating, advising, reporting, directing or supervising, or managing any of the foregoing, that requires the application of engineering principles and that concerns the safeguarding of life, health, property, economic interests, the public welfare or the environment.” Note 2 This definition is circular, defining engineering as applying engineering principles (although the French pages are more specific Note 3). Alberta, Note 4 and Newfoundland and Labrador, Note 5 more specifically define the practice of engineering as “the principles of mathematics, chemistry, physics or any related applied subject” and Prince Edward Island has similar wording, Note 6 whereas Quebec considers the field of practice to include works using “processes of applied chemistry or physics.” Note 7 In practice, most jurisdictions do not formally require an engineer’s oversight for a shielding design, with the exception of Quebec and Ontario. As part of any engineering design work, field reviews are required, which include visual inspections and scatter surveys in Table 5. Consequently, with regards to Table 6, an engineer is not obligated to use only specific design documents permitted by regulations or accreditation agencies, but are expected to use any and all methodologies that would be considered good practice and obvious to peers performing similar design work.

**Table 6:** Presently allowed shielding design methodologies.

Province or territory	NCRP 49 (1976)	BIR (2000)	NCRP 147 (2004)	BIR (2012)	Other (list)/notes
Alberta		X	X		
British Columbia			X	X	BC Centre for Disease Control has some standard templates that can be used.
Manitoba			X	X	
New Brunswick					Unregulated
Newfoundland and Labrador			X	X	CNSC GD‐ 52 used in part for PET/CT and SPECT/CT
Northwest Territories and Nunavut					Not regulated but recommended NCRP 147
Nova Scotia					Unregulated. Nova Scotia Health Authority uses NCRP 147
Ontario	X				Health Canada Safety Code 20A
Prince Edward Island					Unregulated. Health PEI uses NCRP 147
Quebec	X	X	X	X	Safety Codes 30 and 35
Saskatchewan			X	X	The Government of SK has some standard templates that can be used in their Shielding Manual.
Yukon					“Recommended Safety Procedures for Installation and Use” published by the Department of National Health and Welfare. Generally, employers voluntarily comply with current industry best practice

As shown in Table 6, for all provinces with regulations, except Ontario, NCRP147 is identified as the main source of information for the design of x‐ray shielding. In Ontario, assuming a radiographic detector has a certain Pb equivalency as suggested by NCRP 147 has to be approved by the x‐ray inspection service. Many provinces also use BIR2000 and BIR2012 which also provide information on dental, BMD, SPECT/CT, and PET/CT installations. The x‐ray Inspection Service (XRIS) of the Ontario Ministry of Health and Long Term Care has advised that room shielding calculation should use the methodology specified in Safety Code 20A, even though the preamble of Safety Code 35 clearly indicates that it replaces the former code. However, there are two exceptions to this recommendation:

- For the recommended dose limits of ionizing radiation, XRIS is not following the limits published in SC20A (1999 printing), instead the limits published in the 1981 version are followed.
- Figure 2 of SC20A, which plots the attenuation in concrete of x‐rays generated at 50 to 300 kVp, has a typographical error in the labeling of the x‐axis where the thickness of concrete should be in centimeters instead of millimeters.

In the time since the survey was conducted, the province of Ontario has repealed the Healing Arts and Protection (HARP) Act with the Oversight of Health Facilities and Devices Act as part of Bill 160, Strengthening Quality and Accountability for Patients Act.^20^ At the time of writing, the Medical Radiation and Imaging Technology Act (2017) is in the consolidation period and not yet in force. Note 8 The Canadian Organization of Medical Physicists strongly supported the modernization of the Ontario Healing Arts Radiation Protection Act^21^ and contributed as a stakeholder in the Health Quality Ontario report on this topic.^22^

There is a wide range of annual dose constraints used for the design of shielding, as shown in Table 7. For x‐ray workers, where there are regulations, the range is 1 to 50 mSv, and the range for the General Public is 1 to 5 mSv. It is also interesting to note that the constraints and dose limits (Table 4) are often different. An appropriate and conservative approach, and one recommended by the authors of this paper who perform shielding design, is to set a shielding design goal of 1 mSv for all cases, allowing future use of adjacent spaces to change without the need to change shielding, for example, if an office fully occupied by a radiation worker becomes office space for a nonradiation worker (general public).

**Table 7:** Dose Constraints used in the different provinces and territories for radiation protection shielding.

Province or territory	Dose constraints	
	X‐ray worker (mSv/y)	General public (mSv/y)
Alberta	20	1
Alberta Health Services	1	1
British Columbia	1	1
Manitoba	1	1
Newfoundland and Labrador	20	2
New Brunswick	NA	NA
Northwest Territories and Nunavut	50	1
Nova Scotia	5	1
Ontario	50	5
Prince Edward Island	NA	NA
Health PEI	20	1
Quebec	1^a^	1^a^
Saskatchewan	20	1
Yukon	50	NA

a Not directly indicated but explicitly implied by good practice in an engineering sense.

### 4.4 Radiographer occupational exposures 1990–2016

**Figure 1:**
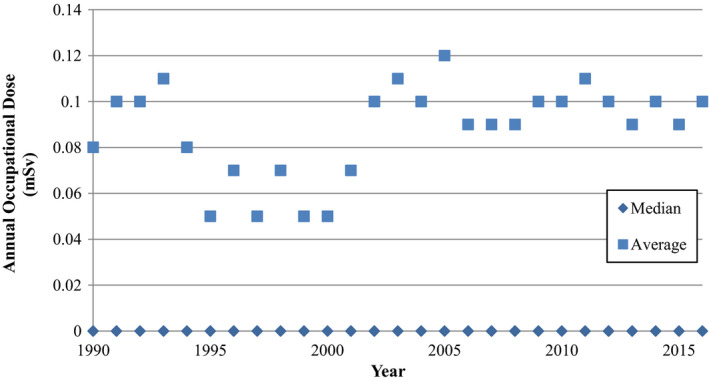
Average and median annual occupational dose for radiographers in Canada. All median values are zero.

The average and median annual occupational dose for radiographic technologists in Canada are shown in Fig. 1. The average value for 2016 is approximately 0.10 mSv/yr and the median value is zero. Technologists working in FGI procedures, who typically experience higher occupational exposures, were not separated from technologists exclusively working in general radiography. The Canadian average is slightly higher than the UK radiographer average value of 0.06 mSv/yr.^6^ It appears that the BIR recommendations to use a dose constraint of 30% of the dose limit (or 0.3 mSv) would also be applicable to Canadian practice, since this constraint level is already achieved, especially considering the measured values reported here include staff who are exposed to workplace radiation without protection from structural shielding, including technologists who work in FGI procedures.

**Figure 2:**
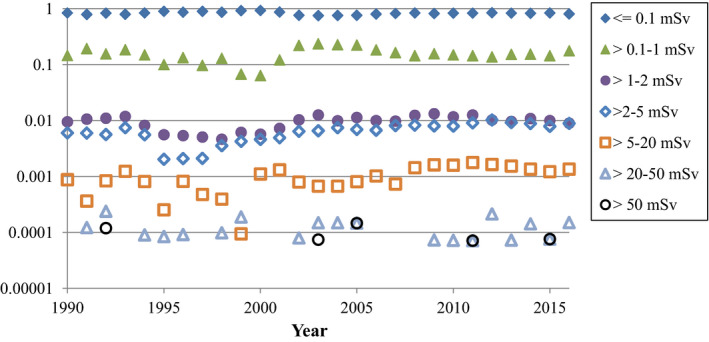
Fraction of Canadian radiography workers receiving different ranges of annual occupational dose. Note the log scale on the *y*‐axis. Years where there were zero workers for a specific range are not shown on the log scale graph.

A breakdown of radiographer occupation exposure by different dose ranges is shown in Fig. 2. The vast majority of workers receive doses below detectability on their badges (represented as <=0.1 mSv on the graph, although newer badges with technology that measures 0.01–0.1 mSv are also included here). The total number of workers whose doses are reported here are 8000 in 1990, increasing to 13 300 by 2016.

While not apparent in the Figures, it is the experience of the authors that general medical imaging technologists rarely register a reading on their dosimeters. In contrast, FGI workers occasionally experience small doses. It is also noteworthy that some nonzero badge readings reported to Health Canada are not accurate in terms of actual staff occupational exposure. For example, if a radiographer has an anomalous reading exceeding 0.25 mSv/quarter on their badge, most jurisdictions will perform an investigation as to the cause. Sometimes the cause is not explained and such higher readings never show up again with that worker. However, since most jurisdictions allow 20 mSv/yr for radiation workers, if the investigators deem it unlikely that the worker's badge readings will exceed this limit for a year even with a high erroneous badge reading, they do not bother issuing a correction to the National Dose Registry. Similar high readings can also be a result of accidental workplace exposure to a group of badges, and again, the investigators might not correct the National Dose Registry records if not doing so has no repercussions to the site or staff member.

An interesting discovery arising from the author correspondence for this work is that some facilities issue a single badge to FGI staff that is to be worn on top of the apron, which is common practice in the United States^23^, ^24^ but uncommon in Canada and not the practice suggested by SC35.^14^ The ICRP recommends two dosimeters be worn—one above the apron at neck level and one under the protective apron for FGI work.^25^ The NCRP recommends both practices but does not recommend a single dosimeter under the apron for FGI work.^24^ For one set of facilities in Canada where they issue a single badge to FGI workers to be worn on top of the apron, we confirmed that from 2008 to 2016, the collar badge readings are being reported as whole‐body readings (occupational dose) with the National Dose Registry. Such practice can routinely result in badge readings exceeding 20 mSv while the true occupational dose is a fraction of this. We verified that at least some of the high “occupational dose” readings in Fig. 2 are not representative of actual occupational dose received by radiography workers.

Another interesting finding is that badging practices vary across Canada. Many jurisdictions require all general duty x‐ray staff to wear a badge in order to demonstrate their workplace exposure does not exceed regulatory limits. In some jurisdictions, since the aforementioned approach showed these staff always received less workplace exposure than what is allowed for the general public (1 mSv/yr), not all staff are routinely badged. A single or handful of full time employees wear dosimeters in order to ensure compliance with dose limits, whereas the rest of the staff wears the dosimeter for the first 6 months and if their workplace exposure is well below 1 mSv/yr they are no longer issued a dosimeter unless the staff member requests it. In such jurisdictions staff working in FGI work still wear dosimeters as do general duty pregnant staff.

**Figure 3:**
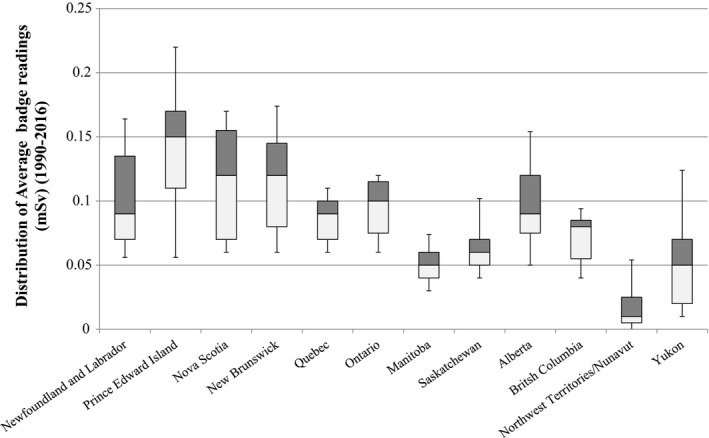
Distribution of average occupational dose badge readings by province/territory for 1990–2016. Box and whisker plot shown with central horizontal line representing the median value, the upper and lower limits of the box are the 25th and 75th percentiles, and the extent of the whiskers are the 10th and 90th percentiles.

Box plot distributions of average occupational dose from 1990 to 2016 by province/territory are shown in Fig. 3, and all median values for each year were reported as zero. However, the true value is more accurately reported as <0.1 mSv, since the minimum reporting values for different badge technologies is 0.1 and 0.01 mSv, and measurements less than these values are reported as zero to Canada’s National Dosimetry Registry.

At the time of writing, Newfoundland and Labrador is presently reviewing their radiation safety legislations and the supporting regulations, whereas Ontario and Manitoba are in the final stages of their processes. Ontario’s higher shielding specifications are based on SC 20A which follows NCRP49 requirements, and Pesianian et al have shown thicker Pb would be specified for shielding designs based on NCRP49 when compared to NCRP147^10^ at greater economic expense. In Newfoundland and Labrador, while not specified in the current regulations, the Minister is referencing Safety Code 35 for inspection standards and exposure limits. There is no explicit distinction between diagnostic x‐ray personnel and nuclear medicine personnel in terms of permissible exposure, but the latter of course are monitored under the CSNC regulations. In practice, it is the experience of these authors that x‐ray radiation workers rarely exceed an occupational exposure of 1 mSv/yr, whereas a nuclear medicine radiation worker has a much higher probability of doing so.

## 5 CONCLUSIONS

In the interests of public safety and to control workplace exposure, it would be useful for different jurisdictions in Canada to adopt a harmonized approach, by implementing uniform dose limits and constraints outlined in Safety Code 35 with the codicil that the design standards are updated to those outlined in NCRP 147 and BIR 2012.

## Conflict of Interest

All authors have no conflict of interest to declare.

## Notes

1
http://laws.justice.gc.ca/eng/regulations/SOR-86-304/page-26.html#h-126 (accessed 2018.12.18)

2
https://engineerscanada.ca/publications/public-guideline-on-the-practice-of-engineering-in-canada#-defining-the-engineering-profession (accessed 2019.04.02, last updated 2012.02)

3
https://engineerscanada.ca/fr/definition-de-lexercice-de-la-profession-dingenieur (accessed 2019.04.02, last updated 2016.04.30)

4
http://www.qp.alberta.ca/documents/Acts/E11.pdf (accessed 2018.12.18, last updated 2015.04.30)

5
https://www.assembly.nl.ca/legislation/sr/annualstatutes/2008/e12-1.c08.htm (accessed 2018.12.18, last updated 2008.12.18)

6
https://www.princeedwardisland.ca/sites/default/files/legislation/E-08-1-Engineering%20Profession%20Act.pdf (accessed 2018.12.18, last updated 2018.06.12)

7
http://legisquebec.gouv.qc.ca/en/ShowDoc/cs/I-9 (accessed 2018.12.18, last updated 2016.01.28)

8
https://www.ontario.ca/laws/statute/17m25 (accessed 2019.04.02, last updated 2017.12.12).

9 Diagnostic Accreditation Program of British Columbia. Accreditation standards 2014 – Diagnostic Imaging. Standard RS6.1.5.
